# A Mixed-Longitudinal Study of Height Velocity of Greek Schoolchildren and the Milestones of the Adolescent Growth Spurt

**DOI:** 10.3390/children9060790

**Published:** 2022-05-27

**Authors:** Kleanthis Kleanthous, Dimitrios T. Papadimitriou, Alexandros Gryparis, Vassiliki Papaevangelou, Anastasios Papadimitriou

**Affiliations:** 1 Third Department of Pediatrics, Attikon University Hospital, National and Kapodistrian University of Athens, 124 62 Athens, Greece; kkleanthousnaxos@yahoo.com (K.K.); vpapaev@gmail.com (V.P.); 2 Endocrine Unit, Second Department of Obstetrics and Gynecology, Aretaieion University Hospital, National and Kapodistrian University of Athens, 124 62 Athens, Greece; 3 Department of Pediatric-Adolescent Endocrinology and Diabetes, Athens Medical Center, 151 25 Marousi, Greece; 4 Department of Speech and Language Therapy, University of Ioannina, 451 10 Ioannina, Greece; alexandros@post.harvard.edu

**Keywords:** height velocity, age at take-off, growth spurt, peak height velocity, total pubertal growth, growth charts

## Abstract

Height velocity (HV) growth charts constructed from longitudinal studies are scarce as they have inherent difficulties, e.g., time, and costs. These difficulties can be partly overcome by a mixed-longitudinal study that covers the entire age range within 3–6 years. To construct HV charts of Greek children and to estimate the milestones of the adolescent growth spurt (AGS), i.e., the onset of AGS (take-off), peak HV, and total pubertal growth (TPG), we performed a mixed longitudinal study in 1514 Greek schoolchildren (6–18 years) with height measurements every 6 months during three schoolyears. We constructed HV charts for boys and girls. Take-off occurs earlier in girls, and, in both sexes, it precedes by 1–1.5 years the appearance of physical signs of puberty. PHV in boys occurs at 12.61 years and in girls at 10.93 years. At take-off, boys are 5 cm taller than girls and TPG for boys is 35.8 cm and for girls 27.3 cm. We constructed HV charts plotted by age, irrespective of pubertal status, and presented data on the milestones of AGS. Furthermore, we suggest that the gradual increase in IGF-1 and E2 that occurs after 5 to 6 years of age triggers the onset of AGS, which precedes physical signs of puberty.

## 1. Introduction

The growth charts used for the assessment of childhood growth, distance and velocity charts, derive from cross-sectional and longitudinal studies, respectively [1]. Most employed are the distance charts, produced by measurements of a substantial number (usually hundreds) of children at each age year. Each measurement depicts height increments from birth until the time of measurement, the natural end of the distance being the final height. However, a more sensitive indicator of childhood growth is height velocity (HV) [2]. While the construction of HV charts requires a smaller number of children, height measurements must be performed every 12 months for lengthy periods of time, ideally from birth until attainment of final (adult) height.

Another way to construct a longitudinal growth chart in a shorter period is with a mixed-longitudinal design [3], which classifies children in age groups of 3 to 6 years apart, with measurements every 6 or 12 months (depending on the study design) [4]. In such studies, within 3–6 years, researchers will have covered the entire age range they aimed to study.

Despite their usefulness in growth assessment, longitudinal studies are scarce [5,6,7,8,9,10,11,12], due to their inherent difficulties, e.g., time, costs, etc. Thus, in clinical practice, HV charts based on studies performed many years ago are still in use, such as the British growth charts, which were constructed in 1976 [13], and in fact, were an improved version of the charts published in 1966 [14]. These charts, however, do not reflect the growth rate of modern children, especially in adolescence, since during the last decades there was a secular trend for earlier sexual maturation in children, particularly in girls [15].

Milestones of the adolescent growth spurt (AGS) are the age of the onset of the growth spurt, i.e., take-off, and peak height velocity (PHV). Moreover, total pubertal growth (TPG) refers to height increment from take-off to final height.

We performed a mixed-longitudinal study that aimed to evaluate the HV of Greek children and, in addition, to estimate the age of the onset of the AGS [“age at take-off” (ATO)], the age and intensity of PHV, and TPG. To our knowledge, this is the first study on the height velocity of Greek schoolchildren.

## 2. Materials and Methods

We conducted a mixed longitudinal study of height and weight in schoolchildren residing in the Greater Athens area, from November 2009 to May 2012. One thousand five hundred fourteen (1514) Greek schoolchildren aged 6–18 years, participated in the study. Measurements concerned four age groups with the first measurement performed on schoolchildren attending the first, fourth, seventh and tenth grades, and the last measurement on schoolchildren attending the third, sixth, ninth and twelfth grades. We measured height every 6 months (November and May) for 2.5 consecutive years. Thus, during the study period, we examined the growth of children of the entire school age range. We performed a total of 7447 measurements of height and weight. Herein, we present data only on height.

Height measurements were performed by a member of our group (K.K.), with a substantial measuring experience prior to the study, using standard anthropometric techniques. Height was measured to the nearest millimeter with a portable wall-mounted KaWe stadiometer (NorEngros, Oslo, Norway). The height of each child was measured three times and the averages of the measurements were recorded.

To estimate the growth rate of each child, we annualized the 6-month measurements in cm/year. We calculated HV for every 6-month age increment, for all ages from 6–18 years, and for each sex, and created sex-specific height velocity charts for children aged 6 to 18 years.

We defined ATO as the age at which the lowest height velocity occurred before the subsequent continual height acceleration that culminated in PHV. The age at PHV was defined as the age at which adolescent acceleration progresses to deceleration, and TPG was calculated by subtracting the height at take-off from the final height.

### Statistical Analysis

Quantitative variables were summarized using mean (SD). For height velocity we used percentiles (i.e., 3rd, 10th, 25th, 50th, 75th, 90th and 97th), referring to the position of a child among a group of normal children; as clinicians often use percentiles as their meaning is more straightforward, especially to the parents. Either way, i.e., with SD or percentiles, we did not note significant differences in growth changes. Qualitative variables were presented using the relative frequency. The quantreg Growth library was used to calculate and design growth curves in the R statistical program. This library implements statistical smoothing techniques using B-splines to estimate nonlinear curves. The corresponding results for the examined variables are presented in the growth charts. Statistical analysis was performed using the statistical program R (R Core Team (2016). R: A language and environment for statistical computing. R Foundation for Statistical Computing, Vienna, Austria. URL: https://www.R-project.org/ (accessed on 27 October 2012). Two-sided *p*-values < 0.05 were considered statistically significant.

## 3. Results

One thousand five hundred and fourteen (1514) schoolchildren were included in the study (809 boys, 705 girls); the participation rate was at least 90% in every measurement. The distribution of the participants in the study by gender according to the grade they attended at the first measurement, is shown in Table 1.

The mean height (SD) and HV (SD) of schoolboys and schoolgirls aged 6–18 years are shown in Table 2.

**Figure 1:**
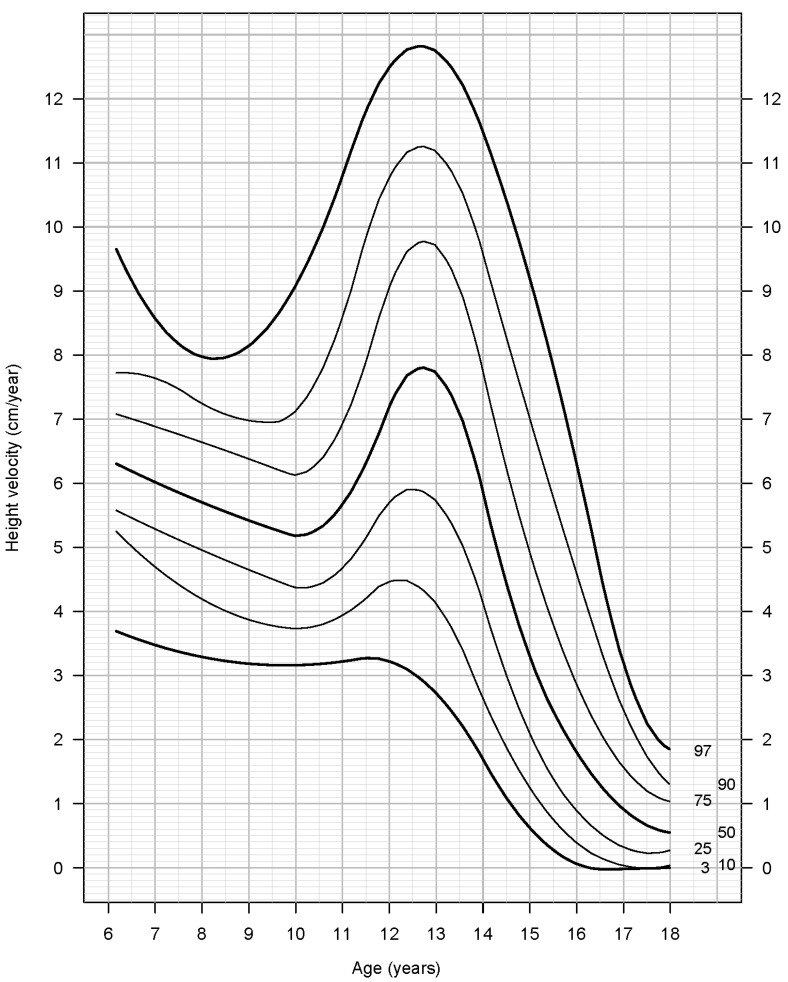
Height velocity for boys.

**Figure 2:**
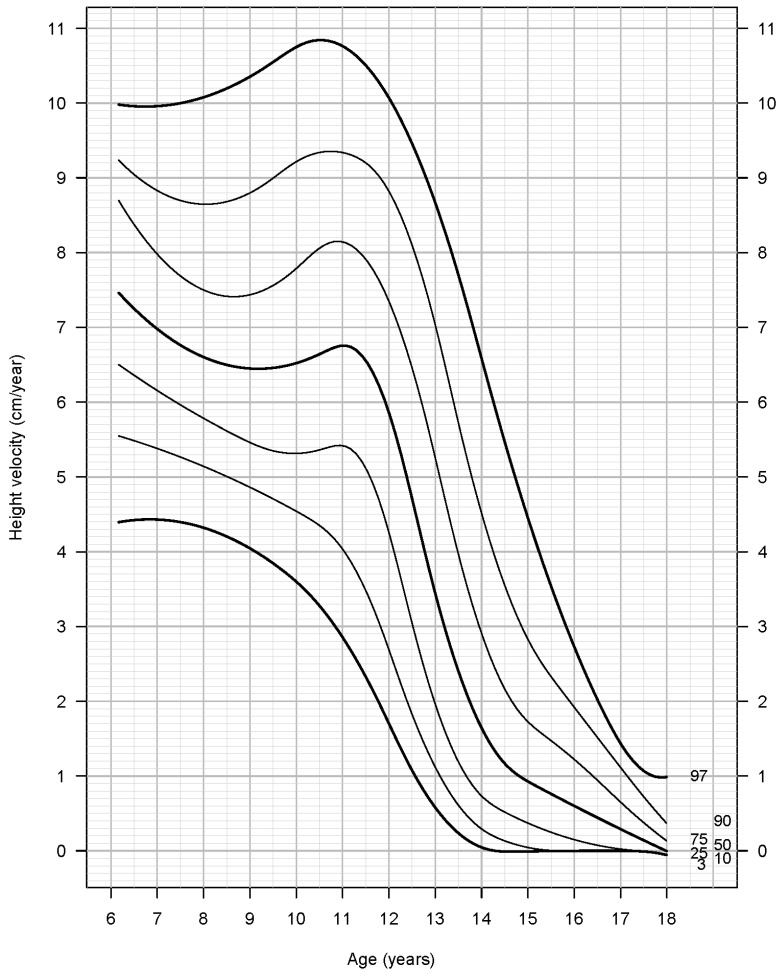
Height velocity for girls.

HV percentiles (3rd, 10th, 25η, 50th, 75th, 90th και 97th) are shown in Table 3 and Table 4, and the HV growth curves are shown in Figure 1 and Figure 2 for boys and girls, respectively.

In boys, after the age of 6 years, a steady decrease in height velocity is observed until the age of 9.86 years (5.18 cm/year), when growth acceleration (take-off) begins, reaching a PHV of 7.82 cm/year at the age of 12.61 years. HV decreases below 1 cm/year (near-final height) after the age of 17 years. Boys from ATO to near-final height gain a total of 35.8 cm, while height gain after PHV is 18.3 cm. Near final height was 176.8 cm.

In girls, HV reaches a nadir at the age of 9.06 years (6.45 cm/year) when take-off begins. PHV occurs at the age of 10.93 years (6.76 cm/year) and HV becomes almost zero (<0.5 cm/year), after the age of 16.5 years (final height). From ATO to final height, girls gain a total of 27.3 cm in height, while height gain after PHV is 16.5 cm. The final height of girls was 163.3 cm.

At ATO the height of boys was 141 cm and of girls was 136 cm. The difference in the TPG between boys and girls was 8.5 cm in favor of the boys.

## 4. Discussion

In this mixed-longitudinal study, we constructed HV growth charts of Greek boys and girls aged 6 to 18 years. Furthermore, we provide data on ATO, PHV and height gain during AGS.

Our study shows that ATO is earlier by one year in girls than in boys. Recent studies in Greek children have shown that the onset of puberty in girls occurs at the age of 10 years [16] and in boys at the age of 11.3 years [17]. Although in this study we did not estimate the participants’ pubertal status, these data suggest that the onset of AGS occurs in girls about 1 year and in boys 1.5 years before physical signs of puberty develop. Relevant studies also report take-off to occur before the development of signs of puberty in both sexes and ATO to be in girls between 9–10 years and in boys between 10–12 years, with considerable variation among different populations [18,19,20,21,22,23], influenced also by secular trend. For example, in six Japanese growth studies published between 1955 and 2000 ATO in boys in 2000 was 1.2 years earlier than in 1955 (8.1 vs. 9.3 years, respectively) (cited in [24]).

The onset of the pubertal growth spurt is triggered by the hormones that regulate growth and development in puberty, i.e., the Growth Hormone/Insulin-like Growth Factor-1 (GH/IGF-1) axis and sex hormones [25]. In the early years of life, up to the age of 5 years in girls and 6 years in boys, IGF-1 levels are rather stable and relatively low; thereafter a gradual increase in IGF-1 is being noted and becomes steeper at the age of 8 years in girls and 9 years in boys [26]. Girls have higher IGF-1 levels than boys until 14 years of age. Estradiol (E2) levels in girls, after infancy, are low but higher than in boys with a slight increase after the age of 7 years and a further increase with age and pubertal stage [27]. These hormonal changes, i.e., increase in IGF-1 and E2 levels, that occur in prepuberty prime children for the development of puberty, physical signs of which are preceded by the onset of the pubertal growth spurt. Thus, higher IGF-1 and E2 levels in girls might drive an earlier onset of puberty. On the other hand, testosterone (T) does not seem to play a direct role in the onset of pubertal growth since in prepubertal boys, T levels are equal or higher than in girls [28] but -despite that- growth spurt in males occurs significantly later than in females.

In the males in our study, the average age of PHV was 12.61 years, whereas for the males in the British study was 14.06 years [29]. For females in our study the average age of PHV was 10.93 years, and for British females was 12.14 years [30]. The girls in our study had an almost constant growth rate for 2 years from take-off to PHV, and thus PHV was not significantly greater than HV at take-off. This may be explained by our previous observation that in Greek girls, the distribution of puberty onset is not Gaussian but skewed to the left [16], meaning that in more girls, breast development occurs earlier rather than later than average. Moreover, other studies, that had HV plotted by age groups, as in our study, showed also a quasi-constant HV from take-off up to PHV [8,31].

The time required from take-off to PHV is 2.75 years for boys and 1.87 years for girls, and from the age of PHV to termination of growth for boys 5.39 years and for girls 5.57 years. Thus, TPG lasts 8.14 years in boys and 7.44 years in girls and during this period boys gain 35.8 cm and girls 27.3 cm in height. It should be noted, however, that the actual duration of pubertal growth in boys is a little longer since in boys we report a near-final height. Fifty-one percent (51%) of TPG of boys and sixty percent of girls occurs after PHV. The near-final height of the boys studied was 176.8 cm and the final height of the girls was 163.3 cm. In a growth study of Greek conscripts performed around the same time as this study, the final height was 178.04 cm [32]. The difference of 13.5 cm from near-final height and 14.74 cm from adult height is in accordance with the literature in which the commonly reported differences in final height between men and women range from 12.5 to 14.5 cm [33,34,35]. The contribution of pubertal growth to final height in boys was 20% and in girls 17%.

At ATO, the height of boys was 141 cm and of girls 136 cm. Therefore, the boys in our study started the adolescent growth spurt 5 cm taller than girls. The difference in TPG between boys and girls was 8.5 cm in favor of the boys. The differences of 5 cm at take-off and 8.5 cm of TPG sum to 13.5 cm and explain the difference in the near-final height of boys and final height of girls.

Height velocity measurements taken at intervals of less than a year could be affected by seasonal variation. However, a large US study of 818 children aged 0–18 years [3], which estimated the growth of children every 6 months, as our study did, found no seasonal effect on the growth rate.

A major strength of our study is the size of the sample since in the study participated 1514 children (809 boys and 705 girls), measured every 6 months for 2.5 years, and in each age group the number of children approximated two hundred with a total of 7447 measurements. Another strength was that all measurements were made by the same experienced examiner minimizing measurement error. A major limitation though, is that it was not possible to physically examine and record the puberty of children during measurements. Therefore, we could not correlate ATO and the age of PHV with the stage of pubertal maturation. Another limitation was that we used the minimum age groups required by a mixed longitudinal study for the classification of the participating children. Some authors suggest that growth measurements should be made more frequently (i.e., every 3 months) at the time of rapid growth [36] to obtain more exact information on growth changes during the estimated PHV period; thus, a limitation of our study is that we evaluated all children every 6 months.

Despite these limitations, our study provides percentiles for evaluating whether the child’s annual growth rate is within the normal range for the population regardless of his/her pubertal status, except in cases of extremely early or late puberty. We suggest that children with abnormally slow or rapid annual growth rates (i.e., <10th centile or >90th centile) are referred for thorough evaluation [37]. In addition, due to the nature of the study, the duration of the pubertal growth of individual children is shorter than that of the population and thus the size of PHV may be underestimated since children with early or late puberty were also probably included.

## 5. Conclusions

In conclusion, we constructed HV charts plotted by age, regardless of the children’s pubertal status. We also estimated the milestones of AGS, i.e., the onset of a growth spurt, PHV and TPG. We found that the onset of the AGS predates the physical signs of puberty by 1–1.5 years. We suggest that AGS is probably triggered by a gradual increase of IGF-1 and E2, which occurs at 5 to 6 years of age, earlier in girls than in boys, priming children for the ensuing development of puberty.

## Figures and Tables

**Table 1:** Distribution of the schoolchildren by gender according to the grade they attended at the first measurement.

Grade	All Children	Gender	Number	Age (Years), Mean (SD)
1st	321	Male	174	6.60 (0.31)
		Female	147	6.42 (0.13)
4th	352	Male	171	9.52 (0.10)
		Female	181	9.46 (0.16)
7th	456	Male	265	12.50 (0.08)
		Female	191	12.46 (0.12)
10th	385	Male	199	15.46 (0.16)
		Female	186	15.41 (0.08)
Total	1514			

**Table 2:** Mean height (SD) and height velocity (SD) of schoolchildren 6–18 years by 6 and 12-month-age increments.

Age, Years	Height (SD) cm	Height Velocity (SD) cm/yr (6 Months)	Height Velocity (SD) cm/yr (12 Months)	Height (SD) cm	Height Velocity (SD) cm/yr (6 Months)	Height Velocity (SD) cm/yr (12 Months)
	Boys			Girls		
6.0	117.7 (4.5)	6.0 (2.2)	6.1 (1.2)	118.0 (5.9)	6.4 (1.3)	6.2 (1.8)
6.5	121.8 (5.3)	6.4 (0.9)		120.9 (5.2)	6.3 (1.6)	
7.0	123.9 (5.6)	6.0 (1.4)	6.1 (1.3)	123.9 (5.7)	6.5 (1.5)	6.3 (1.5)
7.5	127.4 (5.4)	5.9 (1.2)		126.6 (5.7)	5.9 (1.4)	
8.0	129.9 (6.1)	5.6 (1.2)	5.7 (1.2)	129.9 (5.6)	5.7 (1.3)	5.8 (1.3)
8.5	133.3 (5.9)	5.6 (1.2)		132.1 (5.7)	5.8 (1.2)	
9.0	135.3 (6.8)	5.8 (1.0)	5.8 (1.2)	135.8 (5.6)	6.7 (1.6)	6.4 (1.7)
9.5	138.7 (5.6)	5.9 (1.5)		136.9 (5.7)	6.2 (2.0)	
10.0	141.4 (6.5)	5.2 (1.5)	5.4 (1.5)	140.7 (6.3)	6.7 (1.9)	6.5 (2.0)
10.5	144.5 (6.2)	5.5 (1.6)		144.2 (6.2)	6.7 (2.0)	
11.0	146.4 (7.0)	5.7 (1.9)	5.8 (1.9)	147.2 (6.5)	6.8 (1.8)	6.8 (1.8)
11.5	150.2 (7.1)	6.7 (2.1)		150.4 (6.8)	6.6 (1.8)	
12.0	153.1 (7.7)	8.1 (2.8)	7.7 (2.7)	154.0 (6.4)	6.0 (2.4)	6.1 (2.3)
12.5	157.7 (8.3)	8.3 (2.9)		156.0 (6.1)	4.9 (3.0)	
13.0	161.5 (8.5)	7.8 (2.8)	7.7 (2.7)	158.9 (6.3)	3.5 (2.3)	3.7 (2.5)
13.5	165.3 (8.1)	7.3 (2.5)		159.8 (6.2)	2.9 (2.2)	
14.0	168.8 (8.1)	6.1 (2.7)	6.1 (2.8)	161.5 (6.2)	2.0 (1.8)	2.0 (1.8)
14.5	171.3 (7.7)	4.6 (2.5)		161.8 (6.2)	1.6 (1.5)	
15.0	172.7 (7.3)	3.4 (2.0)	3.7 (2.3)	163 (6.7)	1.2 (1.0)	1.1 (1.0)
15.5	173.8 (6.5)	2.9 (2.6)		162.1 (5.9)	0.8 (1.1)	
16.0	174.6 (6.6)	2.2 (2.0)	2.2 (2.0)	162.5 (7.0)	0.8 (0.9)	0.8 (0.9)
16.5	175.1 (5.9)	1.4 (1.2)		163.1 (6.7)	0.6 (0.7)	
17.0	175.7 (6.1)	1.0 (1.0)	1.0 (0.9)	163.1 (7.4)	0.3 (0.4)	0.4 (0.5)
17.5	176.6 (6.4)	0.7 (0.7)		163.2 (7.1)	0.2 (0.3)	
18.0	176.8 (6.0)	0.7 (0.8)	0.8 (0.7)	163.3 (7.1)	0.2 (0.3)	0.2 (0.3)

**Table 3:** Height velocity percentiles of schoolboys 6–18 years old.

Age (Years)	3rd	10th	25th	50th	75th	90th	97th
6	3.5	4.2	5.1	5.4	6.4	8.2	9.5
6.5	5.1	5.4	5.7	6.2	6.9	7.7	8.2
7	3.2	4.3	5.2	6.2	7.0	7.4	8.3
7.5	3.8	4.6	5.2	5.8	6.7	7.6	8.4
8	3.2	4.1	4.8	5.5	6.3	7.1	7.8
8.5	3.7	4.1	4.8	5.6	6.5	7.1	7.4
9	3.7	4.3	5.3	5.9	6.5	7.0	7.5
9.5	4.0	4.2	4.9	5.8	7.0	7.9	9.0
10	2.8	3.6	4.2	5.0	6.1	7.0	7.6
10.5	2.8	3.7	4.6	5.4	6.4	7.2	8.4
11	3.3	3.7	4.3	5.2	6.5	8.2	10.6
11.5	3.5	4.3	5.3	6.4	7.8	9.8	11.5
12	4.3	5.1	5.8	7.7	10.0	11.8	14.2
12.5	3.5	4.7	6.0	8.5	10.6	11.9	13.4
13	2.9	4.3	5.8	7.8	9.7	11.0	13.1
13.5	2.3	4.2	5.4	7.4	9.1	10.6	12.1
14	2.0	2.8	4.1	6.1	7.6	9.9	11.2
14.5	0.9	1.5	2.6	4.2	6.0	8.3	9.8
15	0.6	1.3	2.1	3.2	4.5	5.7	8.1
15.5	−0.2	0.6	1.0	2.2	4.0	6.1	8.5
16	−0.1	0.1	1.0	1.9	3.1	5.3	6.7
16.5	−0.1	0.1	0.5	1.3	2.0	2.8	4.2
17	0.0	0.0	0.3	0.8	1.5	2.3	3.4
17.5	0.0	0.0	0.2	0.5	1.3	1.6	2.1
18	0.0	0.0	0.3	0.6	1.0	1.3	2.6

**Table 4:** Height velocity percentiles of schoolgirls 6–18 years old.

Age (Years)	3rd	10th	25th	50th	75th	90th	97th
6	5.4	5.5	6.4	7.3	8.4	8.7	8.7
6.5	4.5	5.3	6.1	6.8	8.0	8.9	10.6
7	5.1	5.5	6.4	7.3	8.2	9.4	10.3
7.5	4.5	5.0	5.8	6.7	7.5	8.7	9.2
8	4.3	5.0	5.7	6.4	7.3	8.3	9.2
8.5	4.2	5.2	6.0	6.6	7.4	8.1	9.2
9	4.6	5.0	5.8	7.1	8.1	9.0	10.5
9.5	3.2	4.1	5.0	5.7	7.2	8.9	11.1
10	3.7	4.3	5.4	6.5	7.8	9.7	10.6
10.5	3.5	4.4	5.3	6.5	8.1	9.4	11.1
11	3.6	4.5	5.4	7.0	8.1	9.1	10.0
11.5	3.0	4.2	5.4	6.7	7.7	8.7	9.6
12	2.2	2.9	4.2	6.0	7.6	8.9	10.2
12.5	0.5	1.3	2.2	4.8	7.4	8.9	10.6
13	0.3	0.9	1.8	3.0	5.3	6.7	8.0
13.5	0.3	0.7	1.2	2.3	4.0	6.0	7.9
14	0.0	0.2	0.7	1.6	2.8	4.0	6.3
14.5	0.0	0.3	0.5	1.3	2.2	3.3	4.3
15	0.0	0.3	0.5	0.8	1.5	2.5	3.5
15.5	0.0	0.0	0.0	0.8	1.4	2.4	3.3
16	0.0	0.0	0.2	0.6	1.5	2.1	2.5
16.5	0.0	0.0	0.2	0.5	1.0	1.7	2.4
17	0.0	0.0	0.0	0.2	0.4	0.9	1.2
17.5	0.0	0.0	0.0	0.2	0.5	0.6	1.0
18	0.0	0.0	0.0	0.0	0.3	0.6	0.9

## Data Availability

Row data can be found in the Hellenic National Registry of Doctorate Theses at https://freader.ekt.gr/eadd/index.php?doc=46957&lang=el (accessed on 22 January 2020).

## Abbreviations

Height velocity (HV), adolescent growth spurt (AGS), total pubertal growth (TPG), peak height velocity (PHV), age at take-off (ATO), standard deviation (SD), growth hormone (GH), insulin-like growth factor-1 (IGF-1), estradiol (E2), testosterone (T).
